# How to use the Standard Protocol Items: Recommendations for Interventional Trials (SPIRIT) in orthodontic research

**DOI:** 10.1590/2177-6709.27.3.e2220290.oar

**Published:** 2022-07-04

**Authors:** Isabela Coelho NOVAES, Luna Chagas CLEMENTINO, Carlos FLORES-MIR, Leandro Silva MARQUES, Paulo Antônio MARTINS-JÚNIOR

**Affiliations:** 1 Department of Child and Adolescent Oral Health, School of Dentistry, Federal University of Minas Gerais (Belo Horizonte, Minas Gerais, Brazil).; 2 Department of Orthodontics, University of Alberta (Edmonton, Alberta, Canada).; 3 Department of Pediatric Dentistry and Orthodontics, Federal University of Vales do Jequitinhonha e Mucuri (Diamantina, Minas Gerais, Brazil).

**Keywords:** Checklist, Clinical trials, Evidence-based dentistry, Guideline adherence, Orthodontics

## Abstract

**Introduction:**

Clinical trial protocols are essential documents that serve as a basis for research planning. The Standard Protocol Items: Recommendations for Interventional Trials (SPIRIT) statement aimed to increase the transparency and integrity of clinical trial protocols.

**Objectives:**

This paper described the main aspects of the SPIRIT, highlighting the importance of using this guideline in Orthodontics.

**Results:**

The SPIRIT is composed of 33 items and the diagram, which were presented and explained.

**Conclusion:**

The use of the SPIRIT checklist must become essential to increase the transparency and integrity of more reliable and less biased clinical trials in orthodontic research, improving the quality of future publications in this field.

## RESUMO

**Introdução:**

Protocolos de ensaios clínicos são documentos essenciais, que servem como base para o planejamento da pesquisa. As diretrizes do *Standard Protocol Items: Recommendations for Interventional Trials (SPIRIT)* objetivam aumentar a transparência e a integridade dos protocolos de ensaios clínicos.

**Objetivos:**

O presente trabalho descreve os principais aspectos do SPIRIT, destacando a importância do uso dessas diretrizes na Ortodontia.

**Resultados:**

O SPIRIT é composto por 33 itens e 1 diagrama, que foram aqui apresentados e explicados.

**Conclusão:**

O uso do *checklist* SPIRIT deve se tornar essencial, para aumentar a transparência e a integridade de ensaios clínicos na pesquisa ortodôntica, tornando-os mais confiáveis e menos tendenciosos, melhorando, dessa forma, a qualidade das futuras publicações nessa área.

## INTRODUCTION

Clinical trial protocols are essential documents that describe the rationale, aims, methods, ethical issues and dissemination plans of clinical trials, and serve as a basis for research planning.^1^ ^,^ ^2^ All clinical trials, including those performed in the area of Orthodontics, should be based on a complete and transparent protocol. High-quality protocols facilitate assessing adherence to scientific, ethical and safety issues before the beginning of the research; analyzing the conduct and monitoring; as well as checking the results after the completion of the study.^3^ ^-^ ^5^ In addition, a good protocol allows evaluation of the consistency between the final report and the original intention.^6^ As a consequence, the importance of protocols has been emphasized by journal editors, reviewers and researchers in diverse areas.^4^ ^,^ ^7^

**Figure 1:**
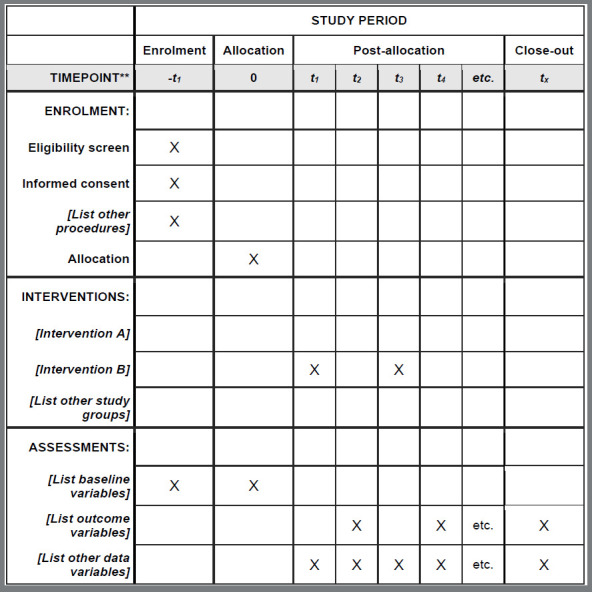
Example template of recommended content for the schedule of enrollment, interventions, and assessments*. Available at: https://www.spirit-statement.org/schedule-of-enrolment-interventions-and-assessments/.

In 2007, a first meeting was held in which a group launched the Standard Protocol Items: Recommendations for Interventional Trials (SPIRIT).^5^ ^,^ ^8^ However, the first article describing this checklist was only published in 2013.^3^ The SPIRIT 2013 Statement was rigorously developed from two systematic reviews and a Delphi consensus process, and involved 115 experts from different areas.^1^ ^,^ ^6^ ^,^ ^9^ SPIRIT adhered to the ethical principles required by the 2008 Declaration of Helsinki, encompassed the items of protocols recommended by the guidance of the International Conference on Harmonisation, Good Clinical Practice E6 guidance, and covered the registration requirements of the World Health Organization, the International Committee of Medical Journal Editors and legislation relating to ClinicalTrials.gov.^7^ The SPIRIT consisted of a list of 33 items and a diagram (Fig 1) and aimed to increase the transparency and integrity of clinical trial protocols, providing evidence-based guidance and facilitating the development of high-quality protocols.^7^ ^,^ ^8^ SPIRIT was not dedicated to prescribe how a study should be designed or conducted, but to help the researcher, including in the area of Orthodontics, to describe clearly and completely what is planned and to understand the main elements of a protocol.^3^

*Recommended content can be displayed using various schematic formats. See SPIRIT 2013 Explanation and Elaboration for examples from protocols.

**List specific timepoints in this row.

Another positive point is that several items from SPIRIT corresponded to items from the Consolidated Standards of Reporting Trials (CONSORT).^10^ ^,^ ^11^ It is important to emphasize that SPIRIT and CONSORT were constructed in a different and complementary way in the development of clinical trials. SPIRIT was developed to assist in the construction, planning and appraisal of protocols for interventional studies, acting as a guide to decrease errors in the design of the study and to avoid possible biases. On the other hand, CONSORT can be used for reporting the results of (already conducted) clinical trials. In addition, it was designed to assist in quality assessment of randomized controlled trials (RCTs) throughout their structure.^11^ Since SPIRIT mirrored applicable items from CONSORT, it became simpler to make the transition from a protocol based on SPIRIT to a final report based on CONSORT.^3^ ^,^ ^5^ The 33 items of SPIRIT were distributed in 5 categories, as follows:

### ADMINISTRATIVE INFORMATION (ITEMS 1-5)

The protocol should present a descriptive title identifying the study design, population and interventions; the trial registration; the protocol version; the date and version identifier; the funding and the roles and responsibilities, when applicable. For example, an adequate title should describe which types of brackets (conventional or self-ligating) were tested in what profile of patients (adolescents) to evaluate what kind of outcomes (arch dimensional changes).

### INTRODUCTION (ITEMS 6-8)

A description of the research question and the justification for conducting the trial should be made considering the benefits and harms for each intervention. The choice of comparators should be explained. The aims and hypotheses should be clarified, and the clinical trial design should be described. For example, a trial comparing two types of treatment for debilitating malocclusions should clearly describe the rationale for conducting the study, explaining the advantages and limitations of each treatment modality, as well as what authors expected to find. 

### METHODS (ITEMS 9-23)

*» Participants, interventions and outcomes (items 9-15):* The researcher should write the study settings and specify eligibility criteria (inclusion and exclusion). For interventions, each study group should present details that allow replication, explain the criteria for interruption/modification of interventions to participants; and present strategies for improving adherence. The protocol should describe all outcomes, including the specific measurement variable, analysis metrics, and method of aggregation. It is essential to inform the recruitment period, interventions, evaluations and visits for participants. It should contain a description of the sample calculation and recruitment strategies of the participants. For example, in a trial comparing the effects of orthodontic treatment on the oral health-related quality of life of adolescent patients, it should describe in details where, when and how recruitment was carried out.
*» Assignment of interventions (for controlled trials) (items 16-17):* The protocol should describe the method used to generate the allocation sequence and the list for stratification factors. It should provide planned restrictions (to reduce the predictability of a randomized sequence) in a separate document, unavailable to those responsible for participant registration. It needs to specify the allocation concealment mechanism used until the assignment of interventions is carried out. Also, the responsible for generating the allocation sequence, registering participants, and assigning participants to the interventions needs to be described. On blinding, the protocol should identify who will be blinded after assignment to interventions and how this will be done. If blind, it is needed to describe the circumstances under which unblinding is permissible, and the procedure for revealing the participant’s intervention during the trial. For example, a trial comparing two drugs for pain control after fixed orthodontic appliance placement should clearly describe how randomization was conducted: using opaque, labeled, and sealed envelopes or trough a website/software. In addition, it should be informed whether a single (patient), double (patient and clinician) or triple (patient, clinician and statistician) blinding was achieved.
*» Data collection, management and analysis (items 18-20):* The protocol should describe evaluation plans for evaluation and outcome collection, baseline, and other trial data, description of study instruments (including reliability and validity), and reference to where data collection forms can be found. The plans for promoting participant retention and complete follow-up, including a list of outcome data of those who discontinue the protocols, should be described. Its should inform plans for data entry, encoding, security, and storage. Reference to the location of information on data management procedures should be included. Statistical methods for analyzing primary and secondary results should also be included. Methods for any additional analyses, definition of the analysis population for non-adherence to the protocol and statistical methods for dealing with missing data should be included. Following the example given above about a trial comparing the effects of orthodontic treatment on the oral health-related quality of life of adolescent patients, it should be described whether the instrument used to measure quality of life of patients was translated and validated for use in the country where the study was carried out, how it is applied (self-applicable or interview) and how scores are interpreted (higher scores denote poorer quality of life?). Data analysis plan, including use of parametric or non-parametric tests, bivariate or multivariate analysis, must be informed.
*» Monitoring (items 21-23):* The protocol should present the composition of the data monitoring committee, the summary of its role, the reporting structure, the statement about its independence from the sponsor and competing interests, and the reference to where more details about the data can be found. It should describe the interim analyses and interruption guidelines. In relation to harm, plans for the collection, assessments, reporting/management of requested and spontaneously reported adverse events and other unintended effects of conducting the trial should be reported. If applicable, it should state the frequency and audit procedures for conducting the trial. Following the example given above about the use of two drugs for pain relief after initial orthodontic treatment, researchers need to clarify how monitoring of side, harmful, undesirable effects would occur and how reporting would be conducted and the protocol for trial interruption.

### ETHICS AND DISCLOSURE (ITEMS 24-31)

The protocol should describe plans for obtaining approval from the Research Ethics Committee/Institutional Review Committee or, if the research is already approved, inform its protocol number. The plans for communicating important protocol changes to relevant parties should be described. It should inform who will obtain consent/assent from prospective trial participants or authorized surrogates, and how it will be conducted. If necessary, it should inform consent for collection and use of data and biological samples from participants. An important point is to describe how personal information of participants will be collected, shared and maintained. The declarations of interest and who will have access to the final trial data set should be presented. It should disclose the contractual agreements that limit this access to the researchers. If they exist, auxiliary care, post-trial and indemnification to the participant who may have suffered damages should be reported. Regarding the dissemination policy, it should mention how the results will be communicated to interest groups, author eligibility guidelines and plans for granting public access to the full protocol, the participant-level data set and the statistical code. For example, in a trial involving evaluation of orthodontic treatment in children and adolescents, it must be stated the obtainment of informed consent from both patients and parents/guardians. 

### APPENDICES (ITEMS 32-33)

The protocol should present the consent form and other documentation related to the participants and authorized surrogates. Plans for collection, laboratory evaluation, and storage of biological samples for genetic or molecular analysis in the current study and for future use should be presented. For example, in a trial that needs to collect saliva, crevicular fluid or blood for biochemical analyses in patients undergoing fixed orthodontic appliance treatment, a document with detailed information about use and storage of these biological samples should be presented. 

SPIRIT also has two extensions, which provide specific guidance for protocols related to patient-reported outcomes (PROs)^2^ and for Traditional Chinese Medicine.^12^ The SPIRIT-PRO extension was developed according to the methodological structure of EQUATOR, and provided international guidance based on consensus about specific PRO information that should be included in clinical trial protocols, such as health-related quality of life or patient-reported symptoms. It consisted of 16 items and aimed to encourage and facilitate the careful planning about the use of PRO components in trials, improving the experimental design.^2^

Following the recommendations of the EQUATOR network, several scientific journals already require that submitted protocol articles should comply with SPIRIT, such as the BMC Oral Health, Medicine (Baltimore), and Trials.^4^ ^,^ ^7^ Interested researchers can use a website (*https://www.spirit-statement.org/trial-protocol-template/*) to create, manage, and register protocols in *clinicaltrials.gov* using the SPIRIT guidance. It should be stated that Brazilians should register trials in the *Registro Brasileiro de Ensaios Clínicos* (ReBEC) (*www.ensaiosclinicos.gov.br*). After submission, the protocols will receive a registration number and will be evaluated by a specific committee. It is important to note that not all items need to be strictly followed. Also, some items can be suppressed, according to trial characteristics.

Both researchers and clinical orthodontists can take advantage of using SPIRIT. Researchers can use SPIRIT to construct high-quality, efficient, and transparent protocols. Clinicians can use SPIRIT to evaluate protocols of clinical trials in orthodontic research. The popularization and application of SPIRIT is indispensable in Orthodontics and should occur as quickly as possible, since an exponential increase in clinical trials is taking place. In conclusion, the development of better and more transparent protocols based on SPIRIT checklist results in ease of conducting the study, reduction of avoidable errors and associated costs, and increases the quality and efficiency of the protocol review. Consequently, these benefits will be translated into higher-quality, more reliable and less biased clinical trials.
